# Absorption mechanism of three curcumin constituents through *in situ* intestinal perfusion method

**DOI:** 10.1590/1414-431X20176353

**Published:** 2017-09-21

**Authors:** Y.-H. Wang, X.-M. Ke, C.-H. Zhang, R.-P. Yang

**Affiliations:** 1 Chongqing Academy of Chinese Materia Medica, Chongqing, China; 2 School of Basic Medical Sciences, Jiujiang University/Key Laboratory of Jiujiang Translational Medicine, Jiujiang, China; 3 Department of Pharmacy College, Southwest University, Chongqing, China

**Keywords:** Curcumin, Demethoxycurcumin, Bisdethoxycurcumin, *In situ* intestinal perfusion, Absorption mechanism

## Abstract

This study aimed to investigate the absorption mechanism of three curcumin constituents in rat small intestines. Self-emulsification was used to solubilize the three curcumin constituents, and the rat *in situ* intestinal perfusion method was used to study factors on drug absorption, including drug mass concentration, absorption site, and the different types and concentrations of absorption inhibitors. Within the scope of experimental concentrations, three curcumin constituents were absorbed in rat small intestines through the active transport mechanism.

## Introduction

Curcumin constituents are the bioactive components (1) of *Curcuma longa* Linn., such as curcuma zedoary, curcuma, turmeric, etc. Curcumin is commonly used as food, natural pigment (2) and cosmetic additives, and possesses extensive pharmacological effects, such as oxidation resistance, anti-inflammation, anti-tumor, the prevention and control of cardiovascular system diseases, etc. (3,4). However, the low solubility of curcumin imposes difficulties in studies *in vivo*. Therefore, in this experiment, self-emulsification was adopted to solubilize the curcumin constituents. *In situ* single pass intestinal perfusion was used to investigate the absorption sites of three constituents in the small intestine, as well as the influence of drug concentrations, concentrations of P-gp inhibitor verapamil hydrochloride, concentrations of MRP2 inhibitor probenecid, and concentrations of energy inhibitor 2,4-dinitrophenol, in order to discuss the absorption mechanism of the constituents and influent factors (5). Drug resistance refers to the insensitivity of target cells to chemotherapeutic agents. Multidrug resistance (MDR) involves a variety of mechanisms, including increases in P-glycoprotein (P-gp), multi-drug resistance-associated protein (MRP), breast cancer resistance protein (BCRP) and lung resistance protein (LRP), decreases in Topo-II activity, elevation of glutathione S-transferase (GST), and alterations of protein kinase C (PKC) function (6).

## Material and Methods

### Instruments, materials and animals

Shimadzu ultrahigh performance liquid chromatograph (LC-30AD dual pump, SPD-20A detector, SPD-M20A detector, LabSolutions chromatographic work station, DGU-20A5R online degasser, SIL-30AC automatic sampler, and CTO-30A column oven; Shimadzu, Japan); BS-224S electronic scale (one hundred-thousandth and one ten-thousandth, Sartorius Stedim Biotech GmbH); DG series card type peristaltic pump (Baoding Lead Fluid Technology Co. Ltd., China).

The following materials were used: bisdemethoxycurcumin reference sample (BDMCur, batch number: FY 11830316, content: 98.3%; Nanjing Zelang Medical Technology Co. Ltd., China); demethoxycurcumin reference sample (DMCur, batch number: FY 11830302, content: 98.0%; Nanjing Zelang Medical Technology Co. Ltd.); curcumin reference sample (Cur, batch number: FY 11830306, content: 98.8%; Nanjing Zelang Medical Technology Co. Ltd.), and curcumin raw material (containing BDMCur 2.44%, DMCur 12.43% and Cur 82.22%; batch number: 120101, Nanjing Zelang Medical Technology Co. Ltd.), characterized by orange color and a slightly bitter odor; polyglycerin oleate (Obleique CC497, French Gattefoss); Tween-20 (Chengdu Kelong Chemical Reagent Factory, China); diethylene glycol monoethyl ether (Transcutol P, (Gattefoss Factory, France); curcumin self-emulsification prescription (Oleique CC497-Tween-20-Transcutol P=0.16:0.54:0.30 (m:m:m); Chongqing Academy of Chinese Materia Medica, China).

Twelve SPF Kunming rats (6 males and 6 females), weighing 200–250 g, were provided by the Laboratory Animal Center, Chongqing Academy of Chinese Materia Medica (Certificate No: SCXK (Yu) 2012–0007).

### Preparation of the test solutions

Calcium chloride, weighing 0.37 g, was dissolved with a small amount of water. Then, 1.40 g glucose, 7.80 g sodium chloride, 0.35 g potassium chloride, 1.37 g sodium bicarbonate, 0.32 g sodium dihydrogen phosphate, and 0.29 g heptahydrate magnesium sulfate were obtained and dissolved in distilled water. This solution was then mixed with the dissolved calcium chloride, pH was adjusted to 6.8 with the diluted hydrochloric acid, and distilled water was finally added to maintain a constant volume of 1000 mL.

Drug-containing self-emulsifying drug delivery system (SEDDS) (drug loading capacity of three constituents: BDMCur 2.6 mg/g, DMCur 15.9 mg/g and Cur 68.5 mg/g; the same below) was weighed by 2104, 1052, 0.526, and 0.264 g, as slightly higher, high, medium and low concentrations. Then, the K-R test solution was added (200 mL at pH 6.8). These were then stirred slightly to realize the emulsification effect. Finally, the perfusion fluid containing the different concentrations of the drug was obtained.

To obtain the P-gp inhibitor verapamil hydrochloride stock solution, verapamil hydrochloride was weighed precisely at 0.1233 g, and placed in a 25-mL volumetric flask. Next, the pH 6.8 K-R solution was added to dissolve and dilute it to the required scale, which was shaken well before use.

Drug perfusion fluid with different concentrations of P-gp inhibitor verapamil hydrochloride were then prepared. Three copies of drug-containing milk (1 g) were weighed precisely, and the verapamil hydrochloride stock solution was added at 0.5, 1.0, and 2.0 mL. Next, the pH 6.8 K-R solution was added to keep the constant volume at 100 mL, and the solution was shaken well. Finally, the drug test solution was obtained with verapamil hydrochloride concentrations of 0.05, 0.1, and 0.5 mmol/L.

To prepare MPR2 inhibitor probenecid stock solution, probenecid was weighed precisely at 0.028 g, and placed in a 25-mL volumetric flask. The methanol solution was then added to dilute the solution to the required scale, and the solution was shaken well before use.

Drug test solutions with different concentrations of MPR2 inhibitor probenecid were prepared. Triplicate drug-containing milk (1 g) was precisely weighed, and the probenecid stock solution was precisely added by 0.5, 1.0, and 2.0 mL. Next, the pH 6.8 K-R solution was added to maintain the volume constant at 100 mL, and the solution was shaken well. Finally, the drug test solution was obtained with a probenecid concentration of 0.02, 0.04, and 0.08 mmol/L, respectively.

To prepare the energy inhibitor 2,4-dinitrophenol stock solution, 2,4-dinitrophenol was precisely weighed at 0.9225 g, and placed in a 25-mL volumetric flask. Then, the acetone solution was added to dilute the solution to the required scale, and the solution was shaken well before use.

Different concentrations of energy inhibitor 2,4-dinitrophenol were then prepared. Triplicate drug-containing milk (1 g) was precisely weighed, and the 2,4-dinitrophenol stock solution was added by 0.5, 1.0, and 2.0 mL. Next, the pH 6.8 K-R solution was added to maintain a constant volume of 100 mL, and the solution was shaken well. Finally, the drug test solution was obtained with 2,4-dinitrophenol at concentrations of 1.0, 2.0, and 5.0 mmol/L.

### Establishment of analytical methodology for constituents in intestinal perfusion fluid

#### Chromatographic conditions

Shim-pack XR-ODS III Chromatographic column, Japan (S/N: 11122273, 2.0 mmi.d×50 mm); flow rate: 0.3 mL/min; column temperature: 30°C; mobile phase: acetonitrile –4% glacial acetic acid (38:62, v/v); detection wavelength: 419 nm.

#### Preparation of the reference solution

An appropriate amount of SEDDS, containing BDMCur, DMCur and Cur reference samples, was obtained with the precise mass. Then, the blank intestinal perfusion fluid was added to prepare the mixed reference sample solution containing 247.0 μg of BDMCur, 679.8 μg of DMCur, and 2,972.0 μg of Cur per 1 mL.

#### Acquisition of blank intestinal perfusion fluid

Healthy SPF Sprague Dawley (SD) rats (2 males and 2 females) were provided by the Laboratory Animal Center, Chongqing Academy of Chinese Materia Medica. Under 12 h fasting status (except for drinking), 20% of urethane solution (5 mL/kg) was injected into the abdominal cavity to anesthetize and immobilize rats. Next, the abdominal cavity was cut along the medioventral line. Then, an incision was made in the upper end of the duodenum and the lower end of the colon, and normal saline was used to wash its contents. Finally, a medical silicone tube was inserted, ligated and fixed, and was connected to a peristaltic pump. After the operation, a degreasing cotton wetted by normal saline was used to cover the wound and insulated by an infrared lamp. Finally, the air was pumped to drain the liquid in the intestinal tract. The pH 6.8 K-R solution was perfused into the intestine for 15 min at 2 and at 0.25 mL/min, with the perfusion fluid collected within 0–3 h. Finally, the blank intestinal perfusion fluid was obtained.

#### Preparation of the test sample solution

An appropriate amount of drug-containing perfusion fluid was obtained and filtered through a 0.22-μm thick filter membrane, and the subsequent filtrate was finally prepared as the test solution.

#### Specificity of the investigation

**Figure 1.**
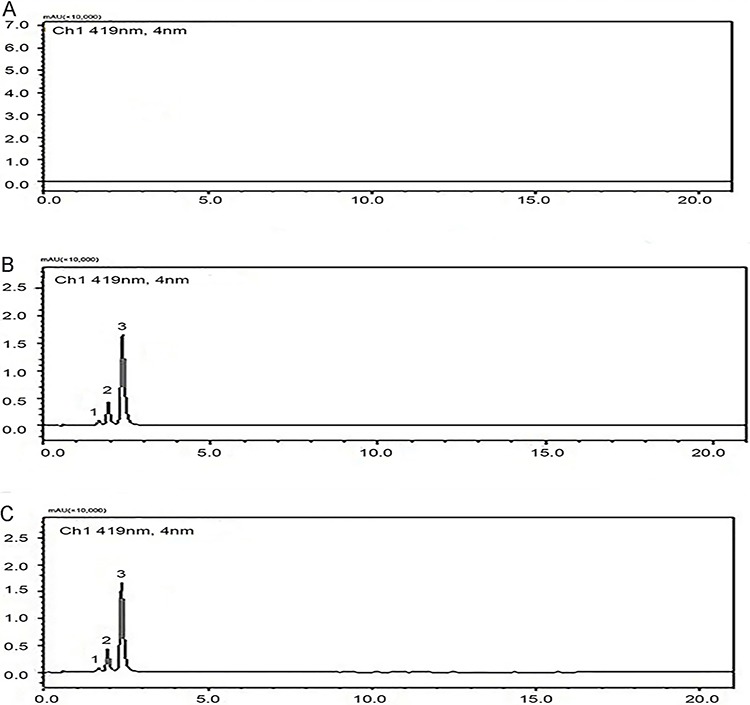
Specificity test results of blank intestinal perfusion fluid (*A*), reference sample solution (*B*) and drug containing perfusion fluid (*C*). 1: bisdemethoxycurcumin (BDMCur); 2: demethoxycurcumin (DMCur); 3: curcumin (Cur).

Ten microliters of blank intestinal perfusion fluid, reference sample solution, and drug-containing perfusion fluid were taken and injected into the high-performance liquid chromatograph. Results are shown in Figure 1, indicating that the separation effect of BDMCur, DMCur and Cur was favorable, and that the blank intestinal perfusion fluid did not interfere with the content measurement of the three constituents.

#### Linear relationship study

**Figure 2.**
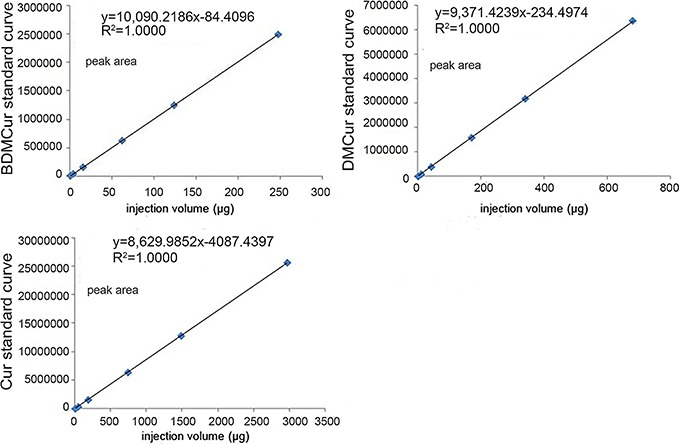
Linear relationship of bisdemethoxycurcumin (BDMCur), demethoxycurcumin (DMCur) and curcumin (Cur).

Firstly, an appropriate amount of the above reference sample solution was taken and diluted into the mixed reference sample solution in a series of concentrations as follows: BDMCur at 0.38, 3.86, 15.44, 61.75, 123.50, and 247.00 μg per 1 mL; DMCur at 1.06, 10.62, 42.49, 169.95, 339.90, and 679.80 μg per 1 mL; Cur 4.64, 46.44, 185.75, 743.00, 1486.00, and 2,972.00 μg per 1 mL. Then, the measurement was carried out according to chromatographic conditions under “2.1”. The linear regression analysis was carried out between the sample size (μg) and the response value peak area by calculating and matching the regression equation and correlation coefficient of the three constituents. Results are shown in Figure 2. Finally, three standard curves were obtained: BDMCur standard curve Y=10090.2x–84.4, r=1.0000; DMCur standard curve Y=9371.4x–234.5, r=1.0000; Cur standard curve Y=8630.0x–4087.4, r=1.0000. These results reveal that the amount of reference sample of BDMCur, DMCur and Cur within the scope of 0.38–247.00, 1.06–679.80, and 4.64–2972.00 μg, respectively, were linearly correlated with the peak area.

#### Precision test

The reference sample solution was taken (containing 123.50 μg of BDMCur, 339.90 μg of DMCur and 1486.00 μg of Cur per 1 mL), and the sample injection was repeated six times. The RSD (%) of the BDMCur, DMCur and Cur peak area were all less than 2%, which revealed that the instrument precision was favorable (Table 1).

**Table 1.** Results of the precision test of bisdemethoxycurcumin (BDMCur), demethoxycurcumin (DMCur) and curcumin (Cur).

Peak area	1	2	3	4	5	6	RSD (%)
BDMCur	1246142	1254365	1257562	1259674	1254544	1256433	0.38
DMCur	3185347	3179976	3199843	3162313	3164323	3139501	0.67
Cur	12824158	12768311	12951145	12896431	12965233	12964831	0.64

RSD: relative standard deviation.

#### Sample recovery rate test

SEDDS with known BDMCur, DMCur and Cur was precisely weighed at 0.01 g, and placed in a 25-mL volumetric flask. The following were then added: 1 mL of reference sample solution containing 0.026 mg/1 mL of BDMCur, 3 mL of reference sample solution containing 0.053 mg of DMCur, 5 mL of reference sample solution containing 0.137 mg of Cur, as well as methyl alcohol, to the required scale, and the solution was shaken well. Finally, the solution was divided evenly into six parts, and a sample injection and measurement was conducted to calculate the content and recovery rate of BDMCur, DMCur and Cur. The average sample recovery rates of BDMCur, DMCur and Cur were 99.66, 99.90, and 99.44%, respectively, and relative standard deviation (RSD) was 1.91, 1.35, and 1.96%, indicating that such method has good accuracy.

In conclusion, this chromatographic condition was applicable to the content measurement of the three constituents (BDMCur, DMCur and Cur) of curcumin SEDDS in intestinal perfusion fluid (Tables 2, 3 and 4).

**Table 2.** Bisdemethoxycurcumin (BDMCur) sample recovery rate test results.

BDMCur (weight/mg)	BDMCur (addition/mg)	BDMCur (measured value/mg)	Recovery (%)	Average recovery (%)	RSD (%)
0.026	0.026	0.052	100.00	100.00	2.44
0.025	0.026	0.052	103.85		
0.028	0.026	0.054	100.00		
0.025	0.026	0.051	100.00		
0.028	0.026	0.053	96.15		
0.024	0.026	0.050	100.00		

RSD: relative standard deviation.

**Table 3.** Demethoxycurcumin (DMCur) sample recovery rate test results.

DMCur (weight/mg)	DMCur (addition/mg)	DMCur (measured value/mg)	Recovery (%)	Average recovery (%)	RSD (%)
0.158	0.159	0.319	101.26	99.90	1.35
0.157	0.159	0.315	99.37		
0.160	0.159	0.320	100.63		
0.154	0.159	0.311	98.74		
0.160	0.159	0.316	98.11		
0.153	0.159	0.314	101.26		

RSD: relative standard deviation.

**Table 4.** Curcumin sample recovery rate test results.

Curcumin (weight/mg)	Curcumin (addition/mg)	Curcumin (measured value/mg)	Recovery(%)	Average recovery (%)	RSD (%)
0.687	0.685	1.374	100.29	99.44	1.96
0.682	0.685	1.337	95.62		
0.680	0.685	1.366	100.15		
0.688	0.685	1.371	99.71		
0.683	0.685	1.376	101.17		
0.684	0.685	1.367	99.71		

RSD: relative standard deviation.

### Stability investigation of curcumin in blank intestinal perfusion fluid

First, 0.25 g (the sample quantity to prepare the high-concentration perfusion fluid) of drug-containing SEDDS was placed in a 25-mL volumetric flask. Then, the blank intestinal perfusion fluid was added to dilute it to the required scale, and shaken well. Afterwards, incubation was carried out at 37.5±0.5°C. Then, samples were taken after 0, 1, 2, 4, and 8 h, and were filtered. Finally, the subsequent filtrate was obtained to carry out the sampling analysis, and the difference of BDMCur, DMCur and Cur from the initial concentration at the different time points was compared. As a result, the RSD values of the three constituents within 8 h in room temperature were less than 2.0%, that is, the various constituents were stable in the blank intestinal perfusion fluid within 8 h (Table 5).

**Table 5.** Stability investigation results of bisdemethoxycurcumin (BDMCur), demethoxycurcumin (DMCur) and curcumin (Cur) in blank intestinal perfusion fluid.

Time (h)	BDMCur (mg/mL)	DMCur (mg/mL)	Cur (mg/mL)
0	0.026	0.159	0.685
1	0.026	0.158	0.684
2	0.026	0.157	0.682
4	0.026	0.156	0.678
8	0.025	0.154	0.668
RSD (%)	1.74	1.23	1.02

RSD: relative standard deviation.

### Adsorption in the intestinal wall and perfusion tube of the constituents

#### Intestinal wall

SPF SD rats were fasted for 18 h (except for drinking). Then, 20% of urethane (5 mL/kg) was injected into the abdominal cavity for anesthesia and immobilization. Afterwards, the abdominal cavity was cut along the medioventral line, and the small intestine was separated. Next, the small intestine was washed with normal saline and cut for approximately 10 cm, of which the mucous layer was routed up with a glass rod and placed into a 50 mL solution containing 27.300 μg of BDMCur, 166.950 μg of DMCur, and 719.250 μg of Cur per 1 mL. This was then incubated for 2 h at 37.5±0.5°C, and the intestinal segment was taken to measure the content of BDMCur, DMCur and Cur in the incubation solution. Finally, its RSD (%) was calculated, and results are shown in Table 6. The RSD (%) of the concentration of the three constituents was less than 2% within 2 h. Hence, it can be concluded that the rat intestinal wall has basically no physical absorption of the drug.

**Table 6.** Results of the absorption test of the intestinal wall by bisdemethoxycurcumin (BDMCur), demethoxycurcumin (DMCur) and curcumin (Cur).

Time (min)	BDMCur (μg/mL)	DMCur (μg/mL)	Cur (μg/mL)
0	27.300	166.950	719.250
30	27.288	165.354	717.346
60	27.247	164.023	716.858
90	27.173	163.724	716.019
120	26.253	162.104	701.751
RSD (%)	1.67	1.11	1.00

RSD: relative standard deviation.

#### Perfusion tube

In order to prevent the absorption of the target composition by the tube, the perfusion tube was soaked with drug-containing intestinal perfusion fluid (containing 27.300 μg of BDMCur, 166.950 μg of DMCur and 719.250 μg of Cur per 1 mL) for 12 h, according to a published process method (7), and then dried. The perfusion fluid flowed through the perfusion tube at 0.25 mL/min and the content of the three constituents at the outlet of the perfusion tube was measured in 0, 30, 60, 90, and 120 min. Afterwards, the content change of the test solution before and after the fluid flowing through the perfusion tube was compared. These results revealed that the RSD (%) of the content change of the three constituents was less than 2.0%. Therefore, the absorption in the perfusion tube for the various constituents can be disregarded (Table 7).

**Table 7.** Results of the absorption test through the perfusion tube of bisdemethoxycurcumin (BDMCur), demethoxycurcumin (DMCur) and curcumin (Cur).

Time (min)	BDMCur (μg/mL)	DMCur (μg/mL)	Cur (μg/mL)
Before perfusion	27.304	166.951	719.253
0	27.263	165.154	717.044
30	27.242	163.423	715.472
60	26.461	162.829	711.751
90	26.156	162.104	693.854
120	27.300	166.950	719.250
RSD (%)	1.90	1.28	1.36

RSD: relative standard deviation.

In summary, the three constituents (BDMCur, DMCur and Cur) of curcumin SEDDS were stable in pH 6.8 K-R blank intestinal perfusion fluid within 8 h, and no obvious absorption occurred in the intestinal wall and perfusion tube.

### Intestinal absorption mechanism of constituents of curcumin SEDDS

The expression of MDRs in the intestine is characterized as follows: P-gp presents the highest expression in colon, followed by distal ileum, and presents less expression in jejunum. MRP2 presents the highest expression in the jejunum segment (8).

#### *In situ* single pass intestinal perfusion operation (7)

Twelve SPF SD rats (6 males and 6 females) were fasted for 18 h (normal drinking). Rats were then anesthetized by injecting 20% urethane solution (5 mL/kg) into the abdominal cavity and immobilized. Next, the abdominal cavity was cut for approximately 3 cm along the medioventral line, and a tube was inserted in the incision of both ends of the intestinal segment to be investigated, which were ligated and fixed. Finally, the intubation tube was connected to the inlet of the 10-cm intestinal segment to be investigated with the peristaltic pump. After the operation, the wound was covered with degreasing cotton wetted by normal saline, and insulation was carried out by the infrared lamp. The small intestine contents were washed with normal saline at 37°C, and were kept stable for 15 min. Afterwards, the air was pumped to drain the liquid in the intestinal tract. Then, the ampoule bottle filled with a known weight of the test solution was used for intestinal perfusion. The perfusion fluid was collected using an EP tube with known weight in the outlet, with the perfusion rate being controlled at 0.25 mL/kg. The timekeeping started when the first drop of the test solution dripped. The perfusion fluid within the first 30 min was ignored, while the test tube filled with the test solution with known weight, and the EP tube that received the perfusion fluid was replaced every 20 min of perfusion; weighing was carried out. At the same time, the weight of the test solution pumped was calculated, and the weight of the effluent (Cout) received was measured every 20 min until the experiment ended after 145 min. Subsequently, the perfusion intestinal segment was cut, its length (l) and perimeter (C) was measured, and the average radius (r) of three perimeters was obtained. After perfusion fluid was filtered through the 0.22-μm thick microfiltration membrane, the subsequent filtrate was obtained and poured into the high performance liquid chromatograph for content measurement. The drug absorption percentage (A%), absorption rate constant (K_a_) and drug apparent absorption coefficient (P_app_) were calculated according to various data, using the following formulas:

$$\text{A\%}=\left(1-\frac{{\text{C}}_{\text{out}}}{{\text{C}}_{\text{in}}}*\frac{{\text{Q}}_{\text{out}}}{{\text{Q}}_{\text{in}}}\right)*100\%$$

$${\text{K}}_{\text{a}}=1-\frac{{\text{C}}_{\text{out}}}{{\text{C}}_{\text{in}}}*\frac{{\text{Q}}_{\text{out}}}{{\text{Q}}_{\text{in}}}*\frac{\text{Q}}{\text{V}}$$

$${\text{P}}_{\text{app}}=\frac{-\text{Q}*\text{In}\left[{\text{C}}_{\text{out}}*{\text{Q}}_{\text{out}}/{\text{C}}_{\text{in}}*{\text{Q}}_{\text{in}}\right]}{2*\pi *\text{r}*\text{I}}$$

where Q_in_ and Q_out_ are the volume of perfusion fluid in the inlet and outlet of the intestinal tract (mL); V is the volume of the perfusion intestinal segment; Q is the perfusion rate; C_out_ and C_in_ are the drug concentrations of the perfusion fluid in the inlet and outlet of the intestinal tract; l and r are the length (cm) and cross-sectional area radius (cm) of the perfusion intestinal segment, respectively.

A%, K_a_ and P_app_ of intestinal segment within five time-periods from 45–65 min to 125–145 min were calculated, and one-way analysis of variance of the results was carried out using the SPSS17.0 statistical software (USA).

#### Test methods

1) The same concentration of drug (group with higher concentration) was used for perfusion at different intestinal segments of the rat to study the absorption site of the drug in the small intestine. Then, A%, K_a_ and P_app_ were calculated. 2) Different concentrations of the drug (higher, high, medium, and low concentration groups) was used for the perfusion experiment in the duodenum segment to study the drug concentration influence on its absorption. 3) The drug that contained different concentrations of P-gp inhibitor verapamil hydrochloride was used for the perfusion experiment on ileum to study the influence of verapamil hydrochloride upon drug absorption. 4) The drug that contained different concentrations of MRP2 inhibitor probenecid was used for the perfusion experiment in the jejunum to study the influence of probenecid upon drug absorption. 5) The drug containing different concentrations of energy inhibitor 2,4-dinitrophenol was used for the perfusion experiment to study its influence upon drug absorption.

## Results

### Experimental results of the intestinal absorption site

One-way analysis of variance results revealed that the absorption difference of BDMCur in the different intestinal segments was significant. The main intestinal absorption site of BDMCur was the duodenum. Compared to the colon, its absorption rate increased (P<0.01) in an extremely significant manner. The absorption of BDMCur in other intestinal segments was not significantly different (P>0.05). The results revealed that the absorption of BDMCur by the upper part of the small intestine of rats was favorable.

The main absorption site of DMCur was the duodenum. Compared with the ileum, the absorption of this intestinal segment increased (P<0.01) in an extremely significant manner. Compared to that of the colon, absorption increased significantly (P<0.05). Moreover, compared with the ileum intestinal segment, the absorption of the jejunum increased significantly (P<0.05). Therefore, the absorption of DMCur in rats mainly concentrated on the upper part of the small intestine, and the order of absorption effect was as follows: duodenum > colon ≈ jejunum > ileum.

**Figure 3.**
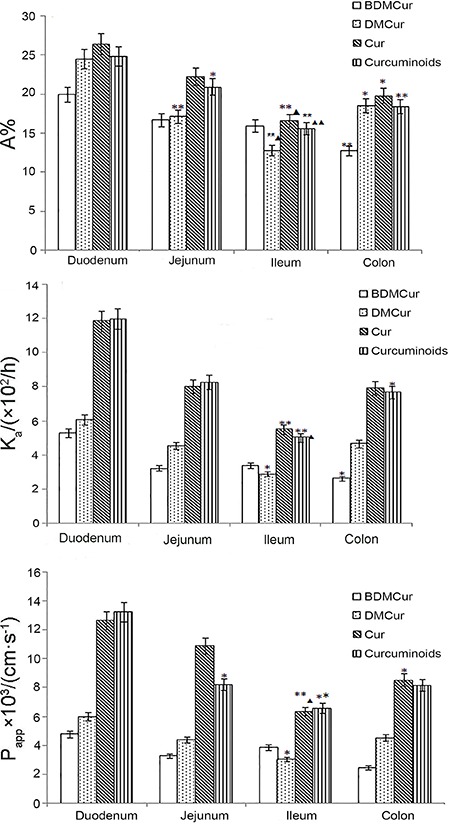
Absorption parameters of bisdemethoxycurcumin (BDMCur), demethoxycurcumin (DMCur) and curcumin (Cur) in various intestinal segments from rats. *Top*: absorption percentage (A%); *middle*: absorption rate constant (K_a_), and *bottom*: drug apparent absorption coefficient (P_app_). *P<0.05, **P<0.01, compared to duodenum; ^▴^P<0.05, ^▴▴^P<0.01 compared to jejunum (ANOVA).

The absorption effect of Cur and curcuminoids in the entire intestinal segment was relatively favorable, while the absorption of the duodenum was the best, followed by the jejunum and colon; the absorption of the ileum segment is relatively poor. The absorption comparison results of the various constituents in the different intestinal segments are shown in Figure 3.

### Absorption of the constituents by the duodenum in curcumin SEDDS with different concentrations

**Figure 4.**
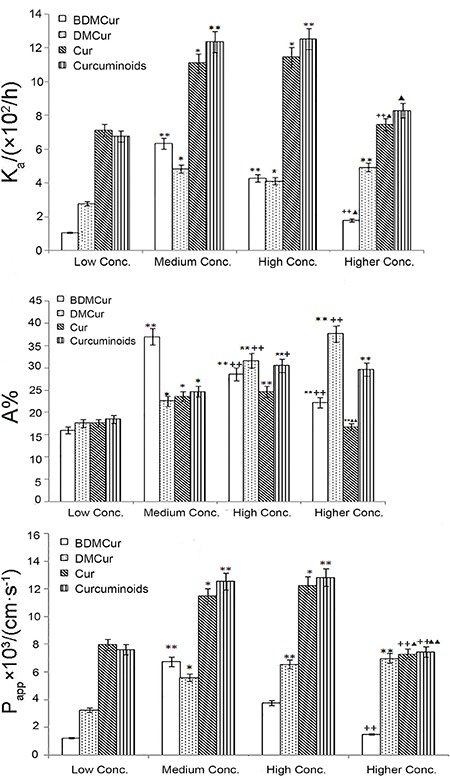
Absorption parameters of bisdemethoxycurcumin (BDMCur), demethoxycurcumin (DMCur) and curcumin (Cur) with different concentrations. *Top*: absorption rate constant (K_a_); *middle*: absorption percentage (A%); and *bottom*: drug apparent absorption coefficient (P_app_). Conc: concentration. **P<0.01, *P<0.05 compared to low concentration; ^++^P<0.01, ^+^P<0.05 compared to medium concentration; ^▴▴^P<0.01, ^▴^P<0.05 compared to high concentration (ANOVA).

Statistical results revealed that A%, K_a_ and P_app_ at different drug concentrations in the small intestine of rats were significantly different. With the concentration scope of 3.43–6.84 μg/mL, A% and K_a_ of BDMCur increased with the increase in concentration, while A%, K_a_ and P_app_ significantly decreased with the further increase in concentration. With experimental concentrations within 20.99–41.82 μg/mL, A%, K_a_ and P_app_ of DMCur increased with the increase in concentration, while A%, K_a_ and P_app_ did not significantly increase in the process when drug concentration increased from 4.83 to 167.27 μg/mL. A%, K_a_ and P_app_ of Cur significantly increased when the concentration increased from 90.42 to 180.12 μg/mL, while with a concentration range of 180.12–360.31 μg/mL, no significant change was found in A%, K_a_ and P_app_. In addition, when the concentration further increased to 720.62 μg/mL, A%, K_a_ and P_app_ did not significantly decrease. The change trend of A%, K_a_ and P_app_ of curcuminoids along with the change in concentration was the same as that of Cur. It can be concluded that the absorption of BDMCur, DMCur and Curcuminoids was better in the duodenum and relatively poorer in the ileum. The concentration inhibiting effect exists in the absorption of BDMCur and Cur, and the saturation phenomenon occurs to the absorption of DMCur. The comparison results of the absorption parameters of various constituents with different drug concentrations in the rat intestines are shown in Figure 4.

### Influence of inhibitor verapamil hydrochloride P-gp on drug absorption

One-way analysis of variance results revealed that P-gp inhibitor verapamil hydrochloride had almost no influence on A% of BDMCur. The medium-concentration and high-concentration inhibitor facilitated a significant increase of DMCur A%. The high-concentration inhibitor caused the A% of Cur and curcuminoids to increase significantly. The high-concentration P-gp inhibitor verapamil hydrochloride significantly accelerated the K_a_ and P_app_ of BDMCur, DMCur, Cur and curcuminoids.

**Figure 5.**
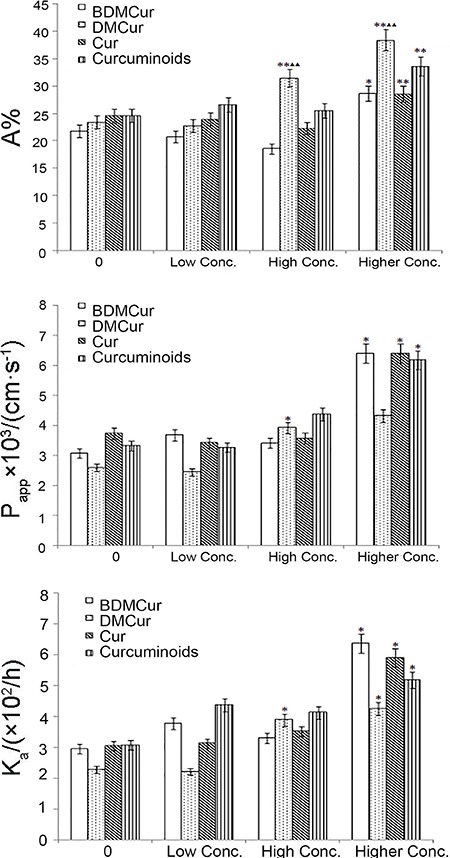
Influence of different verapamil hydrochloride concentrations on absorption parameters of bisdemethoxycurcumin (BDMCur), demethoxycurcumin (DMCur) and curcumin (Cur). *Top*: absorption percentage (A%); *middle*: drug apparent absorption coefficient (P_app_), and *bottom*: absorption rate constant (K_a_). Conc: concentration. **P<0.01, *P<0.05 compared to medicine group without inhibitor; ^▴▴^P<0.01 compared to medicine group with low concentration of inhibitor (ANOVA).

The above results reveal that verapamil hydrochloride can significantly improve the absorption of BDMCur, DMCur, Cur and curcuminoids. Hence, it has a synergic relationship with the drug, that is, drug absorption is influenced by the verapamil hydrochloride transport substrate. Presumably, the absorption mechanism of BDMCur, DMCur and Cur may be the same as that of verapamil hydrochloride. The influence of different concentrations of P-gp inhibitor verapamil hydrochloride upon intestinal absorption of various constituents is shown in Figure 5.

### Influence of MRP2 inhibitor probenecid on drug absorption

Statistical analyses revealed that MRP2 inhibitor probenecid significantly increased the A% of BDMCur, DMCur, Cur and curcuminoids, and the medium-concentration probenecid significantly increased the A% of BDMCur. When probenecid concentration was further increased, the A% of BDMCur barely changed. Furthermore, the A% of DMCur, Cur and curcuminoids increased with the increase in probenecid concentration.

The high-concentration MRP2 inhibitor probenecid significantly increased the K_a_ of BDMCur. The K_a_ of DMCur and Cur increased with the increase in probenecid concentration. The high-concentration of probenecid significantly accelerated the K_a_ of curcuminoids.

Results showed that high-concentration of MRP2 inhibitor probenecid significantly increased the P_app_ of BDMCur, DMCur, Cur and curcuminoids.

**Figure 6.**
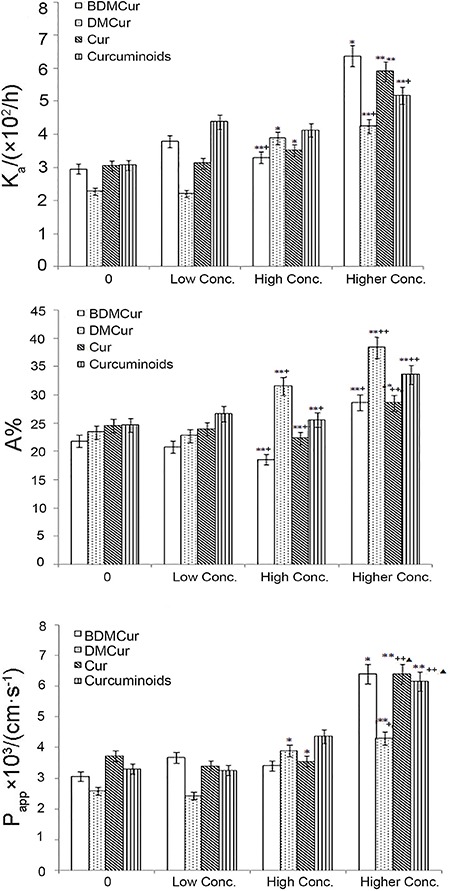
Influence of different probenecid concentrations on absorption parameters of bisdemethoxycurcumin (BDMCur), demethoxycurcumin (DMCur) and curcumin (Cur). *Top*: absorption rate constant (K_a_); *middle*: absorption percentage (A%); and *bottom*: drug apparent absorption coefficient (P_app_). Conc: concentration. **P<0.01, *P<0.05 compared to medicine group without MRP2 inhibitor; ^++^P<0.01, ^+^P<0.05 compared to medicine group with low concentration of MRP2 inhibitor; ^▴^P<0.05 compared to medicine group with high concentration of MRP2 inhibitor (ANOVA).

The above results reveal that the absorption of BDMCur, DMCur and Cur has a synergic relationship with probenecid. Presumably, the absorption mechanism of BDMCur, DMCur and Cur might be the same as that of probenecid. The influence of different concentrations of probenecid upon intestinal absorption for various constituents is shown in Figure 6.

### Influence of energy inhibitor 2,4-dinitrophenol on drug absorption

**Figure 7.**
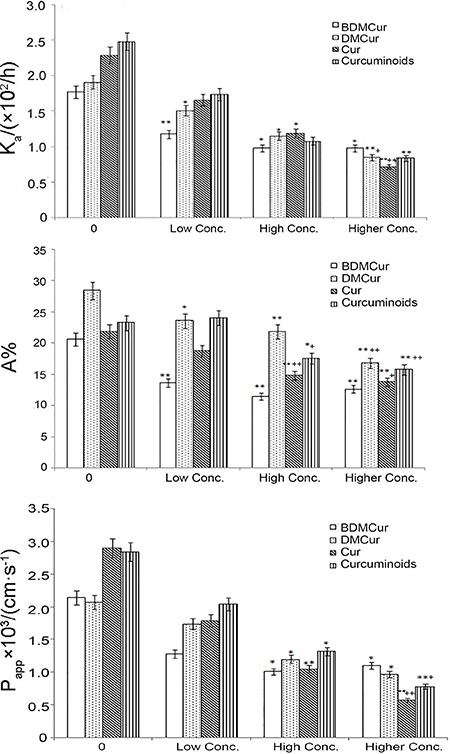
Influence of different energy inhibitor 2,4-dinitrophenol concentrations on absorption parameters of bisdemethoxycurcumin (BDMCur), demethoxycurcumin (DMCur) and curcumin (Cur). *Top*: absorption rate constant (K_a_); *middle*: absorption percentage (A%); and *bottom*: drug apparent absorption coefficient (P_app_). Conc: concentration. **P<0.01, *P<0.05 compared to medicine group without energy inhibitor; ^++^P<0.01, ^+^P<0.05 compared to medicine group with low concentration of energy inhibitor (ANOVA).

Compared with the group without inhibitor, the A% of BDMCur, DMCur, DMCur and curcuminoids of the group with the energy inhibitor decreased in an extremely significant manner (P<0.01). Furthermore, both K_a_ and P_app_ decreased significantly (P<0.05). These indicate that the intestinal absorption process of BDMCur, DMCur and Cur has an energy-dependent action (results are shown in Figure 7).

## Discussion

In the rat *in situ* single pass intestinal perfusion experiment, the small intestine absorbed both drug and moisture, decreasing the volume of perfusion fluid, allowing volume correction. Therefore, the weight correction method was used for correcting the perfusion fluid volume.

We chose to carry out the experiment in the intestinal segment with highly-expressed P-gp and MRP2 in terms of the intestinal absorption influence of P-gp and MRP2 upon BDMCur, DMCur and Cur. It was reported (9) that the expression of P-gp in the jejunum, the far-end ileum, and colon increased successively, but the complicated environment due to a large number of microorganisms and hydrolases in the colon easily influenced the experimental results. Therefore, the far-end ileum segment was chosen as the target intestinal segment for studying the influence of P-gp upon intestinal absorption of the three constituents. Since the expression of the MRP2 in the jejunum segment is the highest (8), the jejunum segment was chosen to study the influence of MRP2 upon intestinal absorption of the three constituents. On the other hand, the absorption effect of the three constituents on the duodenum was favorable. The experiment on the influence of energy upon target composition absorption was carried out in this intestinal segment.

If the P_app_ of the drug in the rat is <3×10^−6^, such drug is difficultly absorbed. If P_app_ is >2×10^−5^, such drug is easily absorbed (10). Therefore, it can be inferred from the experimental data that BDMCur, DMCur and Cur are substances that can be easily absorbed.

The absorption of BDMCur and Cur have concentration-dependent inhibition. The absorption of DMCur has a saturation level, and its absorption mechanism may be carrier-mediated. Further experiments indicated that the participation of verapamil hydrochloride and probenecid could significantly increase the absorption of BDMCur, DMCur and Cur. Therefore, BDMCur, DMCur and Cur may be the substrates of P-glycoprotein and MRP2; the transport mechanism of BDMCur, DMCur and Cur is the active transport in the carrier-mediated transport. P-glycoprotein and MRP2 can pump the substrates from the serosal side to the mucosal side, then into the intestines cavity and discharged, which may lead to a decrease in transmembrane absorption of drugs, decreasing the bioavailability. The reason for the low bioavailability of oral curcumin may be related to its low solubility and the efflux effect of P-gp and MRP2 in intestinal epithelial cells during intestinal absorption. For some drugs that can be identified and excreted by P-glycoprotein and MRP2, their absorption can be improved by inhibiting the expression of P-glycoprotein and MRP2, which improve the bioavailability. It is suggested that during clinical medication, the solubility of curcumin can be increased, and it can be concurrently used with P-gp substrate inhibitor verapamil and/or MRP2 substrate probenecid to improve the oral bioavailability of drug and increase its clinical efficacy.

The main chemical constituents of curcumin include bisdemethoxycurcumin, demethoxycurcumin and curcumin. These constituents are similar in structure, are insoluble in water, and have many similar pharmacological activities, such as anti-inflammation, antioxidation, blood lipid regulation, liver protection, gallbladder strengthening, among others. However, each has its own advantage: curcumin has the strongest anticancer activity, demethoxycurcumin has the most potent hypolipidemic effect, and bisdemethoxycurcumin is good for bile and has a potent inhibitor effect on the growth of endothelial cells. The curcumin constituents have broad pharmacological effects; however, they are easily affected by temperature, humidity, light and pH value. Their instability seriously restricts their clinical application.
